# Association between Atopic Dermatitis and Colorectal Cancer: TET2 as a Shared Gene Signature and Prognostic Biomarker

**DOI:** 10.7150/jca.92238

**Published:** 2024-01-20

**Authors:** Zhi-Qing Zhan, Ze-Min Huang, Rui-Qi Zeng, Yu-Hua Luo, Zhi-Xin Xie, Ying-Zhou Chen, Pei-Zhen Chen, Tian-Ye Luo, Baoqing Sun, Zhangkai J. Cheng

**Affiliations:** 1 Department of Clinical Laboratory, National Center for Respiratory Medicine, National Clinical Research Center for Respiratory Disease, State Key Laboratory of Respiratory Disease, Guangzhou Institute of Respiratory Health, The First Affiliated Hospital of Guangzhou Medical University, Guangzhou Medical University, Guangzhou, China.; 2 Division of Gastroenterology and Hepatology; Shanghai Institute of Digestive Disease; NHC Key Laboratory of Digestive Diseases; State Key Laboratory for Oncogenes and Related Genes; Renji Hospital, School of Medicine, Shanghai Jiao Tong University, Shanghai, China.; 3 Department of Clinical Medicine, Guangzhou Medical University, Guangzhou, China.; 4 Department of Gastroenterology and Hepatology, West China Hospital, Sichuan University, China.

**Keywords:** Mendelian randomization, atopic dermatitis, colorectal cancer, TET2, WGCNA.

## Abstract

**Background:** Recent studies have linked atopic dermatitis (AD) to colorectal cancer (CRC) risk. Their causality and potential molecular mechanisms remain unclear.

**Methods:** We performed Mendelian randomization (MR) analysis to evaluate the causality between AD and CRC. Summary statistic data-based Mendelian randomization (SMR) analysis was used to identify CRC-related causal genes. Transcriptome analyses and immunohistochemical methods were applied to investigate the shared gene signature and potential mechanisms that contribute to the pathogenesis of both AD and CRC. A predictive analysis was performed to examine the shared gene signature associated with immunotherapy response in CRC.

**Results:** MR analysis indicated a causal association between AD and a decreased risk of CRC. SMR analysis uncovered TET2 as a CRC-related causal gene, showing an inverse relationship with the risk of CRC. Transcriptome analyses identified TET2 as a shared gene signature between AD and CRC. Decreased TET2 expression is associated with impaired demethylation and worse prognosis in CRC patients. We observed ten pathways related to the inflammatory response and immune regulation that may be shared mechanisms underlying both AD and CRC. These findings were validated through single-cell analysis. TET2 shows promise as a powerful predictive biomarker for cancer prognosis and immunotherapy response in CRC.

**Conclusion:** There is a causal association between AD and a decreased risk of CRC. AD may influence the occurrence of CRC by modulating immune and inflammatory responses. TET2 could serve as a potential biomarker for prognosis and may be considered a novel therapeutic target for methylation and immune-related interventions.

## Introduction

The association between atopy and cancer has been a subject of ongoing debate, with conflicting opinions on whether atopy acts as a risk factor or a protective factor. One hypothesis posits that the heightened immune response associated with atopy may have a suppressive effect on the development of cancer cells. On the other hand, chronic inflammation is also recognized as a potential contributing factor to the risk of developing certain types of cancers 1, 2. Atopic dermatitis (AD), a prevalent chronic inflammatory skin condition, is characterized by an impaired skin barrier and aberrant immune function, with a global prevalence of approximately 10% among adults and 20% among children 3. Studies examining the relationship between AD and cancers yielding very different results included hematologic malignancy, melanoma, pancreatic cancer, ovarian cancer, esophageal cancer, and prostate cancer 4-6. These seemingly conflicting theories may be reconciled, and their applicability may vary depending on the specific type of cancer. Colorectal cancer (CRC) poses a significant global health challenge, being the second most common cause of cancer-related mortality and the third most prevalent cancer globally 7. However, prior population-based investigations examining the correlation between AD and CRC have shown inconsistent findings 8-10. In addition, these studies faced methodological challenges, including medical surveillance bias, low statistical power, and potential confounding effects of AD drug usage, which could lead to misleading findings. Thus, the relationship between AD and CRC remains uncertain.

**Figure 1:**
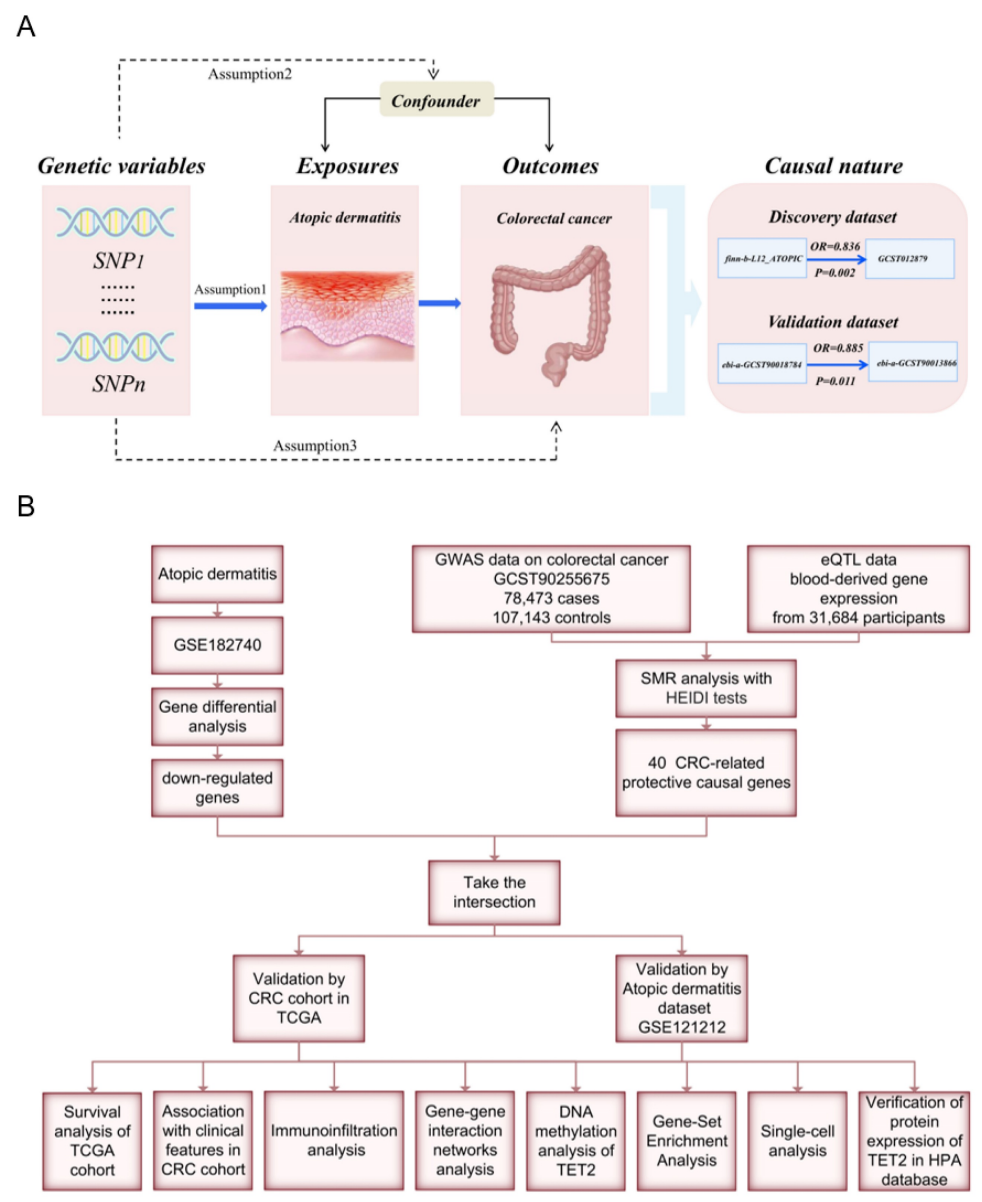
Workflow of this study design. (A) Mendelian randomization revealed the causality between atopic dermatitis and colorectal cancer. (B) Bioinformatic analyses revealed the shared genes between atopic dermatitis and colorectal cancer.

Mendelian randomization (MR) analyses employ genetic variants as instrumental variables (IVs) to evaluate the causal relationship between risk factors and outcomes. By utilizing disease-independent genotypes, this approach effectively addresses confounding and reverse causality biases 11. In this study, we aim to address the following issues: Is there a causal association between AD and CRC? If so, what are the underlying mechanisms and shared gene signature contributing to both diseases? We performed an MR analysis to investigate the link between AD and CRC. Weighted gene co-expression network analysis (WGCNA) and summary statistic data-based Mendelian randomization (SMR) analysis were used to identify the shared gene signature whose expression levels have causal effects on CRC, followed by validation though protein immunohistochemical staining. Furthermore, we employed multiple bioinformatic analyses to investigate the functions and prognostic value of the identified shared hub genes. A flow chart of this study design is presented in Figure 1.

## Materials and Methods

### MR analysis evaluating the causality between AD and CRC

#### Data Source and Selection of Instrumental Variables (IVs)

To enhance the reliability of our findings, we examined the association between AD and CRC using both discovery and validation datasets. Genome-wide association study (GWAS) data on CRC of European ancestry were obtained from the GWAS catalog (https://www.ebi.ac.uk/gwas/). Specifically, the CRC GWAS datasets GCST012879 and ebi-a-GCST90013866 were used as the discovery outcome dataset and validation outcome dataset, respectively. To avoid bias caused by sample overlap, we used data from nonoverlapping datasets for different exposure-outcome pairs. GWAS data on AD of European ancestry were retrieved from the IEU Open GWAS Project (https://gwas.mrcieu.ac.uk/). Detailed information on the GWAS data is shown in Table S1.

We selected IVs based on the following assumptions: (1) IVs should be strongly associated with exposures; (2) IVs should not be related to confounders; and (3) IVs should not be directly associated with outcomes 11. To meet the first assumption, we selected SNPs associated with each trait at the genome-wide significance threshold of p < 5 × 10^-8^. Only SNPs with a long physical distance (≥10000 kb) and less possibility of linkage disequilibrium (R^2^ < 0.001) were retained.

### Statistical methods

Multiple MR approaches were utilized to investigate the causal relationship between AD and CRC. These approaches included the inverse variance weighted (IVW) method, MR‒Egger, weighted median, weighted mode, and simple mode 11. Additionally, the presence of potential directional pleiotropy in the genetic variants was assessed using the MR‒Egger intercept's test, and heterogeneity was evaluated through Cochran's Q test 11. Statistical significance was determined at a threshold of P < 0.05.

### SMR analysis to identify CRC-related causal genes

SMR analysis has been described by Zhu et al. in detail 12. In short, SMR utilizes MR principles to simultaneously investigate GWAS and eQTL summary statistics to assess the association between gene expression and a trait attributable to a shared variant at a specific locus. In this study, SMR analysis was employed to identify novel causal genes related to CRC and explore their potential functional significance. CRC GWAS data (id: GCST90255675) were used for SMR analysis. The eQTL data were obtained from the eQTLGen consortium (https://eqtlgen.org/phase1.html). It is widely acknowledged that the eQTL effects observed in blood tissue can serve as a proxy for eQTL effects in the most pertinent tissues associated with diverse traits or diseases 12. In our study, we employed summary-level data for blood-derived gene expression from a cohort of 31,684 individuals of European ancestry. This dataset included 25,482 samples from whole blood and 6,202 samples from peripheral blood mononuclear cells, serving as the eQTL data for our analysis.

To distinguish between the pleiotropy (or causality) model and the less biologically relevant linkage model, we utilized the heterogeneity in dependent instruments (HEIDI) test. This was crucial in ensuring that the significant SMR results were indeed indicative of pleiotropy or causality rather than being influenced by the less relevant linkage model 12. The SMR and HEIDI tests were employed to investigate the enrichment of cis-eQTLs in the context of CRC. This approach aimed at determining whether the effect size of genetic variants impacting the risk of CRC is mediated by gene expression, thereby prioritizing genes associated with these gene sets for subsequent functional studies. The genome-wide significance level for the SMR test was P_SMR_ < 2.3×10^-5^ (0.05/2131, Bonferroni correction). A p value threshold of P_HEIDI_ > 0.01 was considered conservative for genes exhibiting no heterogeneity. SMR analysis was implemented through SMR software.

### Bioinformatic analyses

#### Data download and processing

To obtain gene expression profiles for AD and CRC patients, we searched the GEO database using relevant keywords and selected specific datasets: GSE182740, GSE121212, and GSE60709 for AD; GSE39582 and GSE113513 for CRC. Differential expression analysis between the AD and control groups was conducted using the GSE182740 dataset. The statistical screening criteria were set as |logFC|≥2 and adjusted p < 0.05. The AD dataset GSE121212 from the GEO database and the CRC cohort from The Cancer Genome Atlas (TCGA) database were used for validation. The workflow is illustrated in Figure 1. Table S2 provides detailed information on the above datasets.

#### Identification and prognostic value evaluation of the shared gene signature

We performed SMR analysis to identify CRC-related causal protective genes (β_SMR_<0 and P<0.05). We then intersected these genes with the DEGs of AD. The resulting genes were considered shared genes between CRC and AD. Nonpaired and paired tissues from the CRC cohort in the TCGA database and the AD dataset GSE121212 were utilized for validation. Shared genes that still exhibited differential expression after validation were defined as the shared gene signature between AD and CRC, which was used for subsequent analyses. We investigated the correlation between the expression of shared gene signature and patient prognosis in CRC by analyzing overall survival (OS), disease-specific survival (DSS), and progression-free interval (PFI) using Kaplan‒Meier (KM) curves generated with the "survminer" and "survival" packages. The necessary data for analysis were obtained from the TCGA database. The Wilcoxon rank sum test was utilized to explore the relationship between the expression of the shared hub gene and clinical features in CRC.

#### Single-Cell Sequencing Analysis and Immune infiltration analysis

The Tumor Immune Single-cell Hub 2 (TISCH2) database (http://tisch.comp-genomics.org/) 13 provides detailed cell-type annotation at the single-cell level, enabling in-depth exploration of the tumor microenvironment. We utilized the GSE146771 dataset and leveraged the TISCH2 database to analyze the correlations between the expression of the shared hub gene and infiltrating immune cells. CIBERSORT can quantify immune cell compositions and identify 22 human hematopoietic cell types in different cancer types using bulk tissue gene expression profiles obtained from RNA sequencing data 14. In this study, we used the CIBERSORT algorithm to analyze immune cell infiltration characteristics in the AD dataset GSE121212 and CRC dataset GSE113513.

#### Gene‒gene interaction networks and DNA methylation analysis of the shared hub gene

GeneMANIA (http://genemania.org/) 15 is a valuable database used to identify and predict proteins with similar functions and potential interactions with key genes of interest. We utilized GeneMANIA to explore the interactions between the shared hub gene and its associated genes. To explore the association between the shared hub gene and DNA methylation, we analyzed HM450 methylation data sourced from the UCSC Xena database (https://xenabrowser.net/). 16 Using the R package "ggplot2," we visualized the methylation differences between tumor and normal samples. Additionally, we utilized the "survminer" R package to construct Kaplan‒Meier curves and assess the correlation between the methylation of the shared gene signature and the prognostic value of the tumor, determined by OS.

#### Gene set enrichment analysis (GSEA)

GSEA was performed on the AD dataset GSE60709 and the CRC dataset GSE39582, respectively. The shared pathways between AD and CRC, as well as the potential mechanisms underlying the reduced risk of CRC in individuals with AD, were identified by overlapping the results from these analyses. The samples were divided into two groups based on the median level of gene expression, and GSEA was conducted on these gene sets using GSEA software. Specifically, we utilized the gseKEGG function of the clusterProfiler package to perform GSEA on hub genes. Filtering criteria were applied, with a q-value less than 0.25 and a p. adj less than 0.05.

#### Protein expression of the shared gene signature by immunohistochemistry

The Human Protein Atlas (HPA) database (https://www.proteinatlas.org/) 17 offers valuable resources such as protein expression profiles, subcellular localization information, and immunohistochemistry images. We obtained immunohistochemical staining images of the shared hub gene from the HPA database. These images provide a visual representation of the differential expression and spatial distribution of the shared hub gene in colon adenocarcinoma and normal colon tissue, as well as rectal adenocarcinoma and normal rectum tissue.

#### Predictive potential of TET2 in CRC immunotherapy response

The immunotherapy response predictive analysis of TET2 was conducted using the CAMOIP (http://www.camoip.net/) 18. To explore the profiles of gene mutation and driver mutation in different TET2 mutation statuses, we analyzed the 20 genes with the highest mutation frequency in CRC patients in the ICI-cohort and TCGA cohorts, respectively. Additionally, we calculated the differences in tumor mutation burden (TMB), neoantigen loads, and MANTIS score between TET2 mutant and wild-type groups in the TCGA-CRC cohort.

## Results

### Results of MR analysis and SMR analysis

The results of the MR analysis are presented in Table S3. For discovery datasets, MR analysis showed that genetically predicted AD causally decreased the risk of CRC (OR = 0.836, 95% CI = 0.748 - 0.936, p = 0.002). In sensitivity analyses, the MR‒Egger intercept test showed no evidence of unbalanced pleiotropy (P _intercept_ = 0.440). Cochran's Q test showed insignificant heterogeneity (P _heterogeneity_ = 0.289). Consistent findings were replicated in the validation datasets, indicating a significant association between genetically predicted AD and a decreased risk of CRC (OR = 0.885, 95% CI = 0.805-0.973, p = 0.0114). The MR‒Egger intercept test revealed no indications of unbalanced pleiotropy (P _intercept_ = 0.071). Cochran's Q test indicated no presence of heterogeneity (P _heterogeneity_ = 0.279).

The SMR analysis revealed a total of 71 genes that exhibited significant associations with CRC, and these genes were identified as CRC-related causal genes (Table S4). Notably, TET2 demonstrated an inverse association with CRC risk (OR_SMR_ = 0.5033, P_SMR_ = 1.54×10^-5^), suggesting a protective role of TET2 in the pathogenesis of CRC.

### Results of bioinformatic analyses

#### TET2 serves as the shared gene signature and CRC prognosis biomarker

**Figure 2:**
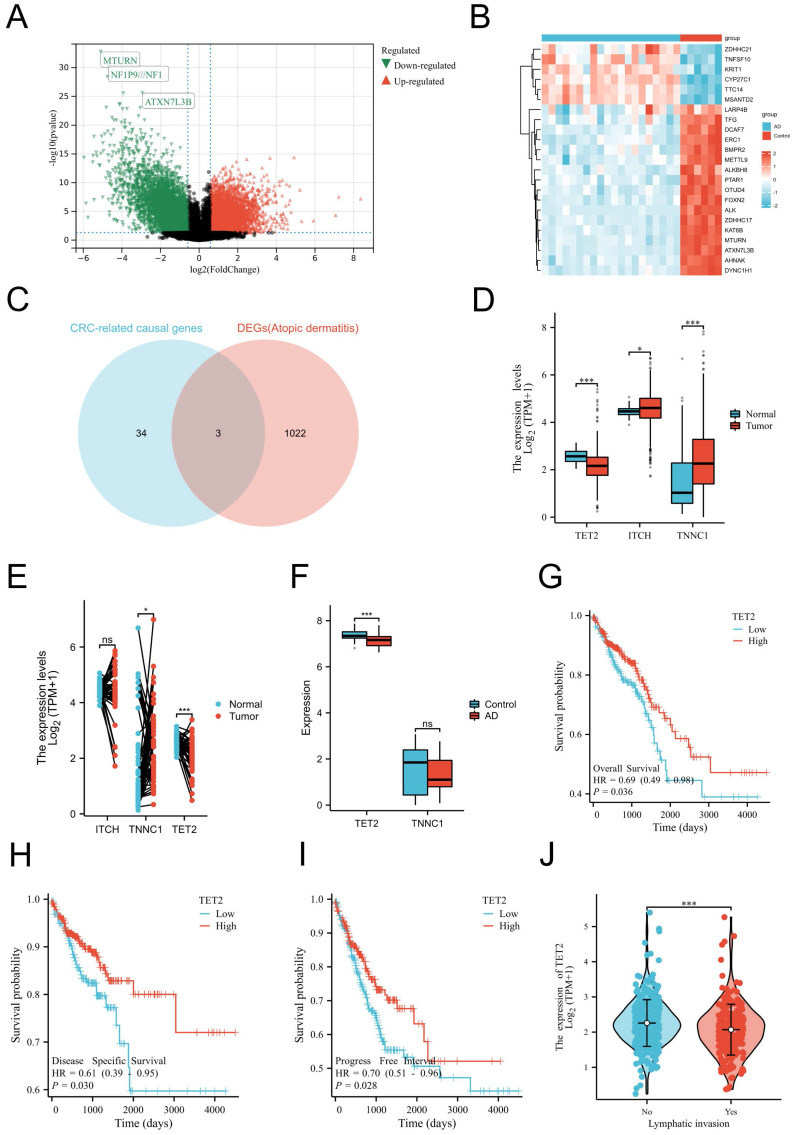
Identification, validation and evaluation of the shared hub gene TET2. (A) Volcano maps of DEGs in the AD dataset GSE182740. The volcano graphs show the DEGs expression distribution. Based on adj. P < 0.05 and |logFC| ≥2 cutoff criteria; red dots indicate up-regulated genes, while green dots represent the down-regulated genes. (B) Heatmaps of DEGs in GSE182740. The heatmaps display the top 23 up-regulated down-regulated genes in the dataset. (C) Intersection of CRC-related protective causal genes and AD down-regulated genes of AD. (D) Validation of shared causal genes in nonpaired samples from the CRC cohort in TCGA. (E) Validation of shared causal genes in paired samples from the CRC cohort in TCGA. (F) Validation of shared causal genes in the AD dataset GSE121212. (G) Overall survival. (H) Disease-specific survival. (I) Progression-free interval. (J) Lymphatic invasion status.

As mentioned before, our MR analyses suggest that AD was causally associated with decreased CRC risk. Thus, we aimed to identify the shared gene that showed protective effects on CRC. CRC-related protective causal genes obtained from SMR analysis and the downregulated DEGs of AD were intersected to identify. The AD dataset GSE182740 identified a total of 2472 DEGs. Among these DEGs, 652 genes were upregulated, while 1025 genes were downregulated, as depicted in Figure 2A. The expression profiles of the top 23 upregulated and downregulated DEGs are presented in Figure 2B. By intersecting the CRC-related protective causal genes obtained from SMR analysis with the downregulated DEGs of AD, we identified three shared genes: ITCH, TNNC1, and TET2, as shown in Figure 2C. The validation of the shared genes was conducted using a cohort of CRC samples from TCGA (Figure 2D-2E) and AD datasets GSE121212 (Figure 2F). Among the shared genes examined, TET2 consistently exhibited differential expression across the validation process and was thus identified as the shared hub gene between AD and CRC. Notably, downregulation of TET2 expression was observed in CRC tissues. Prognostic evaluation of TET2 was conducted through survival analysis. The hazard ratios (HRs) of TET2 for OS, DSS, and PFI were calculated as 0.69 (95% confidence interval [CI]: 0.49-0.98, p = 0.036), 0.61 (95% CI: 0.39-0.95, p = 0.03), and 0.7 (95% CI: 0.51-0.96, p = 0.028), respectively. These results suggest that lower expression of TET2 is associated with a worse prognosis for CRC (Figures 2G-2I). Additionally, a significant correlation was observed between lymphatic invasion and lower levels of TET2 expression (p < 0.05) (Figure 2J).

#### Methylation of cg09666717 and cg12306086 is associated with poorer CRC prognosis

**Figure 3:**
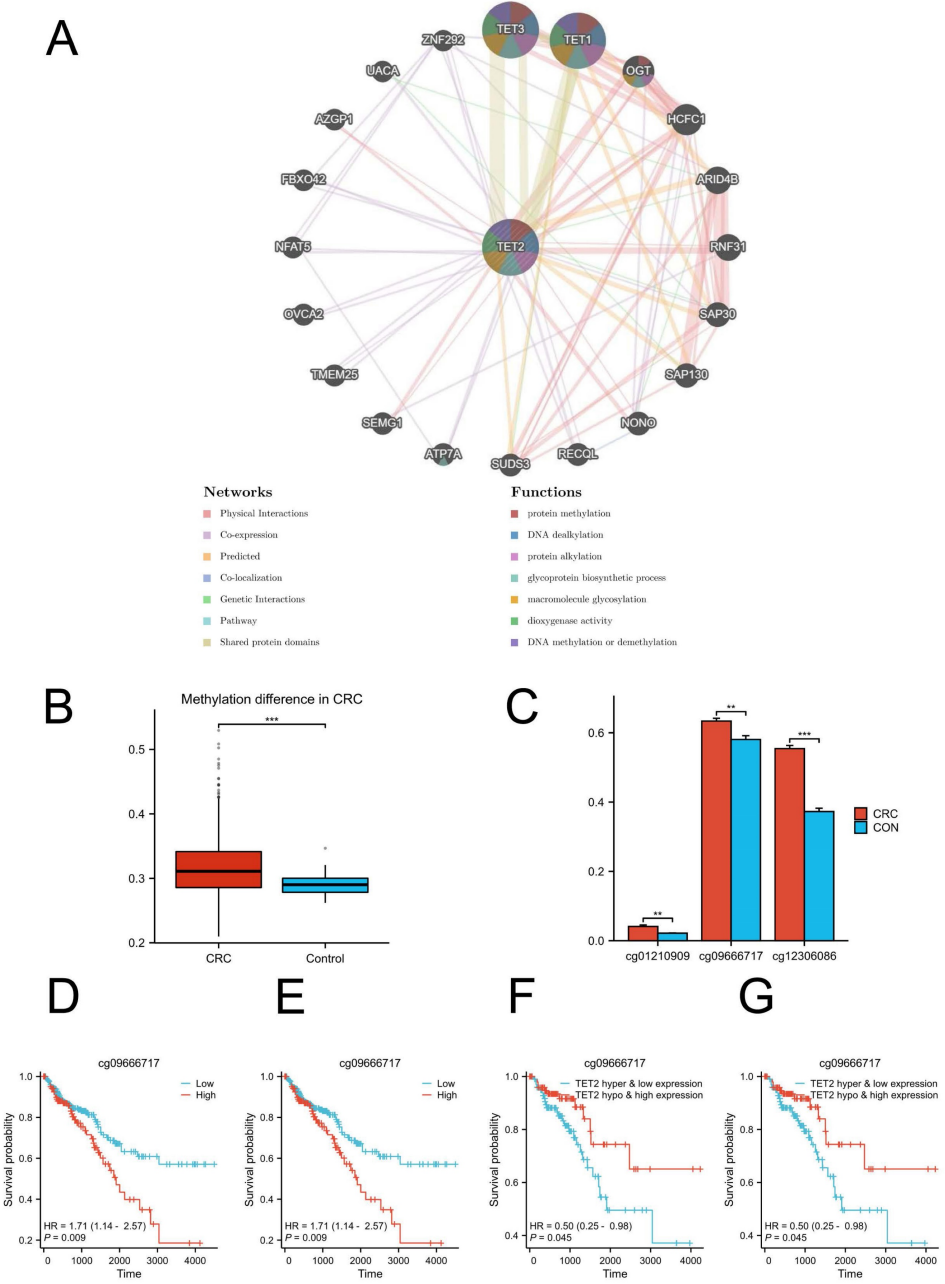
(A) Results of GeneMANIA showing the interactions between TET2 and its related genes. (B) Methylation difference in CRC. (C) Hypermethylated sites of TET2. (D) Overall survival is lower in breast cancer patients with high cg09666717 methylation. (E) Overall survival is lower in breast cancer patients with high cg12306086 methylation. (F) The high cg09666717 methylation and low TET2 expression group showed lower overall survival. (G) The high cg12306086 methylation and low TET2 expression groups showed lower overall survival.

GeneMANIA analysis revealed the genes that exhibited significant interactions with TET2 (Figure 3A). The closest interacting molecules with TET2 were TET1, TET3, and O-linked N-acetylglucosamine transferase (OGT). The biological functions of TET2 and its associated genes are primarily associated with DNA methylation or demethylation, protein methylation and alkylation, and macromolecule glycosylation. Then, we performed TET2 DNA methylation analysis. TET2 showed significant methylation upregulation in CRC compared to controls (Figure 3B).

The analysis of differential methylation sites between tumor and normal samples revealed that cg01210909, cg09666717, and cg12306086 exhibited hypermethylation in TET2 (Figure 3C). Notably, hypermethylation of cg09666717 and cg12306086 were both significantly associated with poor prognosis. The hazard ratios (HRs) for cg09666717 and cg12306086 were calculated as 1.71 (95% CI: 1.14-2.57, p = 0.009) and 1.98 (95% CI: 1.24-3.14, p = 0.004), respectively (Figure 3D-3E). In addition, CRC patients with both cg09666717 site hypermethylation and low TET2 gene expression had a significantly increased survival probability compared to the hypermethylation combined with high TET2 gene expression group. Similar findings were observed in the analysis of the cg09666717 site (Figure 3F-3G).

#### Potential mechanisms involving TET2 contribute to the reduced risk of CRC in AD patients

**Figure 4:**
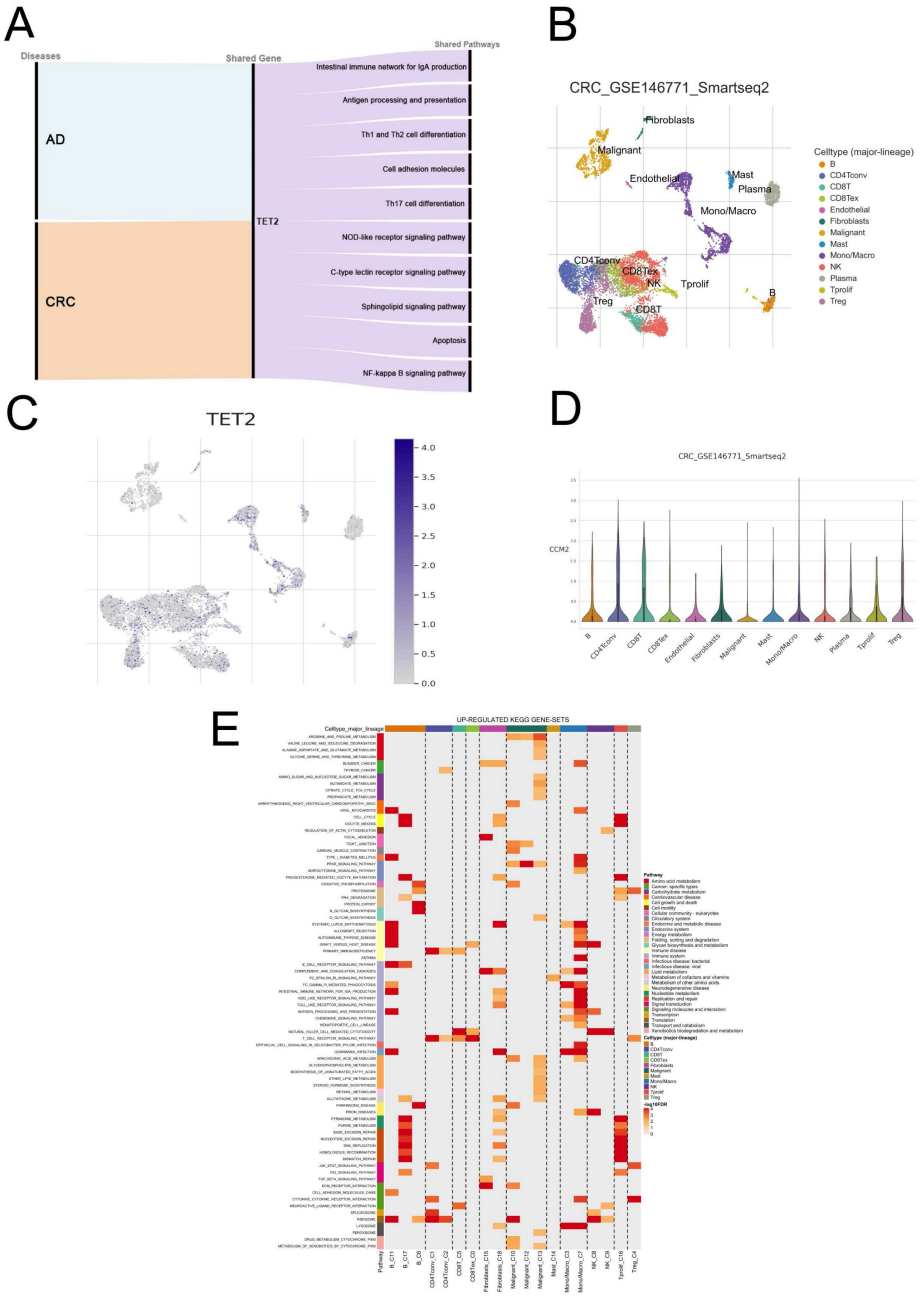
Biological processes and single-cell analysis results associated with TET2. (A) The shared signaling pathways between atopic dermatitis and CRC in the low TET2 expression group. (C) UMAP of fifteen cell types in GSE146771. (D-E) TET2 had high expression levels in macrophages. (F) Upregulated KEGG gene sets.

GSEA was employed to explore the shared pathways between AD and CRC, as well as the potential mechanisms underlying the reduced risk of CRC in individuals with AD. The detailed results can be found in Tables S5-6. In both AD and CRC, the high TET2 expression group exhibited enrichment in ten pathways associated with inflammatory response and immune regulation, including intestinal immune network for IgA production, antigen processing and presentation, Th1 and Th2 cell differentiation, cell adhesion molecules, Th17 cell differentiation, NOD-like receptor signaling pathway, C-type lectin receptor signaling pathway, sphingolipid signaling pathway, apoptosis, and NF-kappa B signaling pathway (Figure 4A; Figure S1).

#### Correlations between TET2 expression and immune cell markers

**Figure 5:**
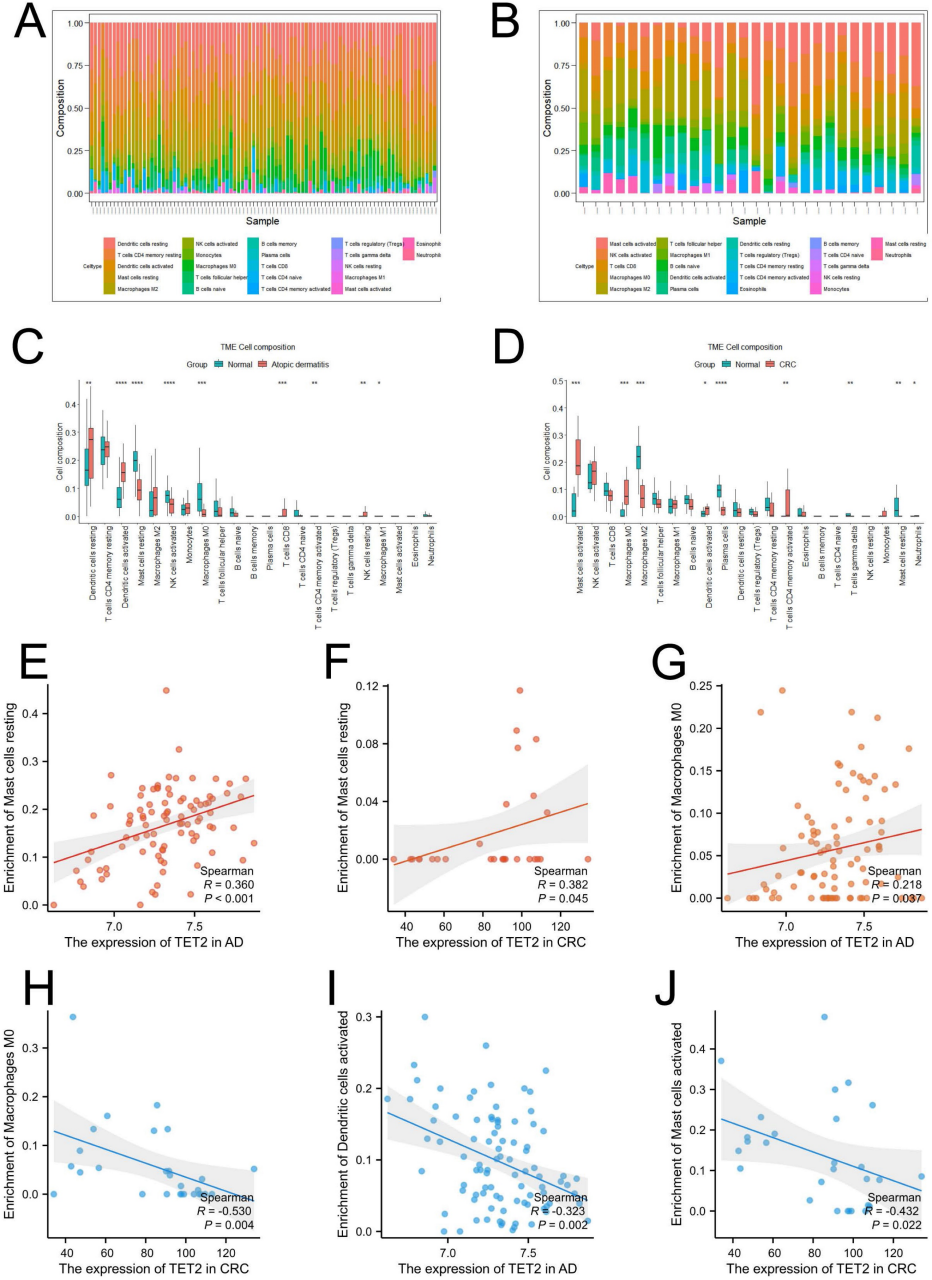
Visualization of immune cell infiltration. (A) Infiltration of 22 types of immune cells in individual samples in the CRC dataset GSE113513. (B) Infiltration of 22 types of immune cells in individual samples in the atopic dermatitis dataset GSE121212. (C) The difference in immune infiltration proportions between the CRC and control groups. (D) The difference in immune infiltration proportions between the atopic dermatitis and control groups. (E-D) Correlation between TET2 and resting mast cells in AD. (H-J) Correlation between TET2 and resting mast cells in CRC.

Single-cell analysis was performed using CRC samples from the TISCH2 database to examine the expression of the hub gene in immune cells associated with the tumor immune microenvironment (TME). In the GSE146771 dataset, a total of 15 cell types were identified (Figure 4B). Figures 4C and 4D illustrate that TET2 exhibited high expression in macrophages. Furthermore, Figure 4E highlights the upregulated pathway gene sets in immune cells, including antigen processing and presentation and cell adhesion molecules, which were primarily enriched in B cells. The NOD-like receptor signaling pathway was found to be activated in macrophages. The CIBERSORT algorithm was employed to analyze immune cell infiltration in the AD dataset GSE121212 and the CRC dataset GSE113513. The results are presented in Figures 5A-5D. In AD patients, TET2 expression exhibited a positive correlation with resting mast cells (Spearman's r = 0.360, p < 0.001) and M0 macrophages (Spearman's r = 0.218, p = 0.037) but was negatively associated with activated dendritic cells (Spearman's r = -0.323, p = 0.002) (Figure 5E-5G). In CRC patients, TET2 expression showed a negative correlation with M0 macrophages (Spearman's r = -0.530, p = 0.004) and activated mast cells (Spearman's r = -0.432, p = 0.022) but was positively associated with resting mast cells (Spearman's r = 0.382, p = 0.045) (Figure 5H-5J).

#### The protein immunohistochemical staining results of TET2

**Figure 6:**
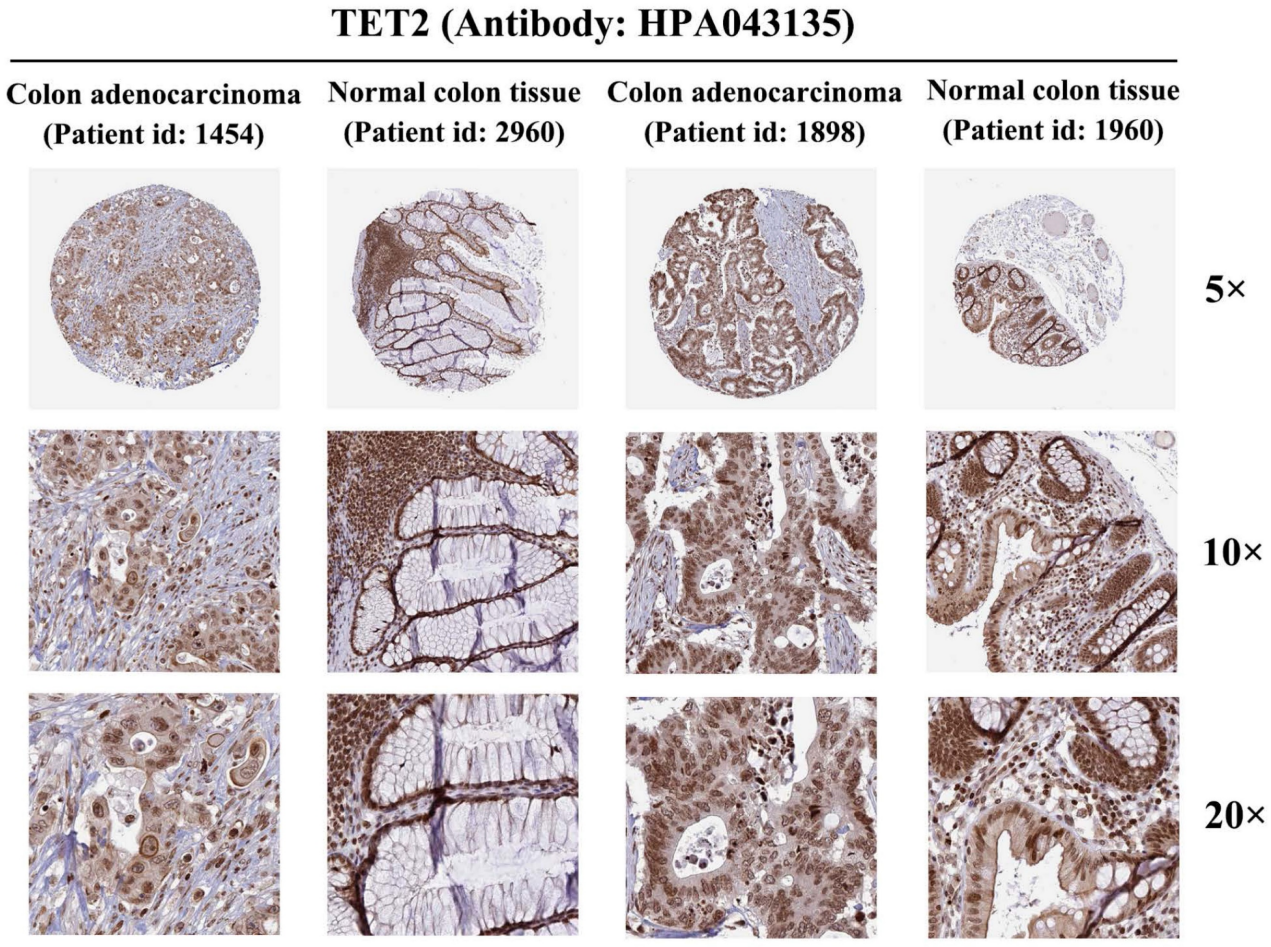
Protein expression of TET2 in colorectal cancer and normal colorectal tissues (antibody HPA 039812).

To further investigate TET2 expression at the protein level, we analyzed colon adenocarcinoma, normal colon tissue, rectal adenocarcinoma, and normal rectum tissue using data from the HPA database. Immunohistochemical staining of TET2 was performed using the antibody HPA 039812. The results obtained from immunohistochemical staining were consistent with the previous transcriptional level observations, thereby providing further validation of the reliability of TET2 (Figure 6).

#### Predictive potential of TET2 in CRC immunotherapy response

**Figure 7:**
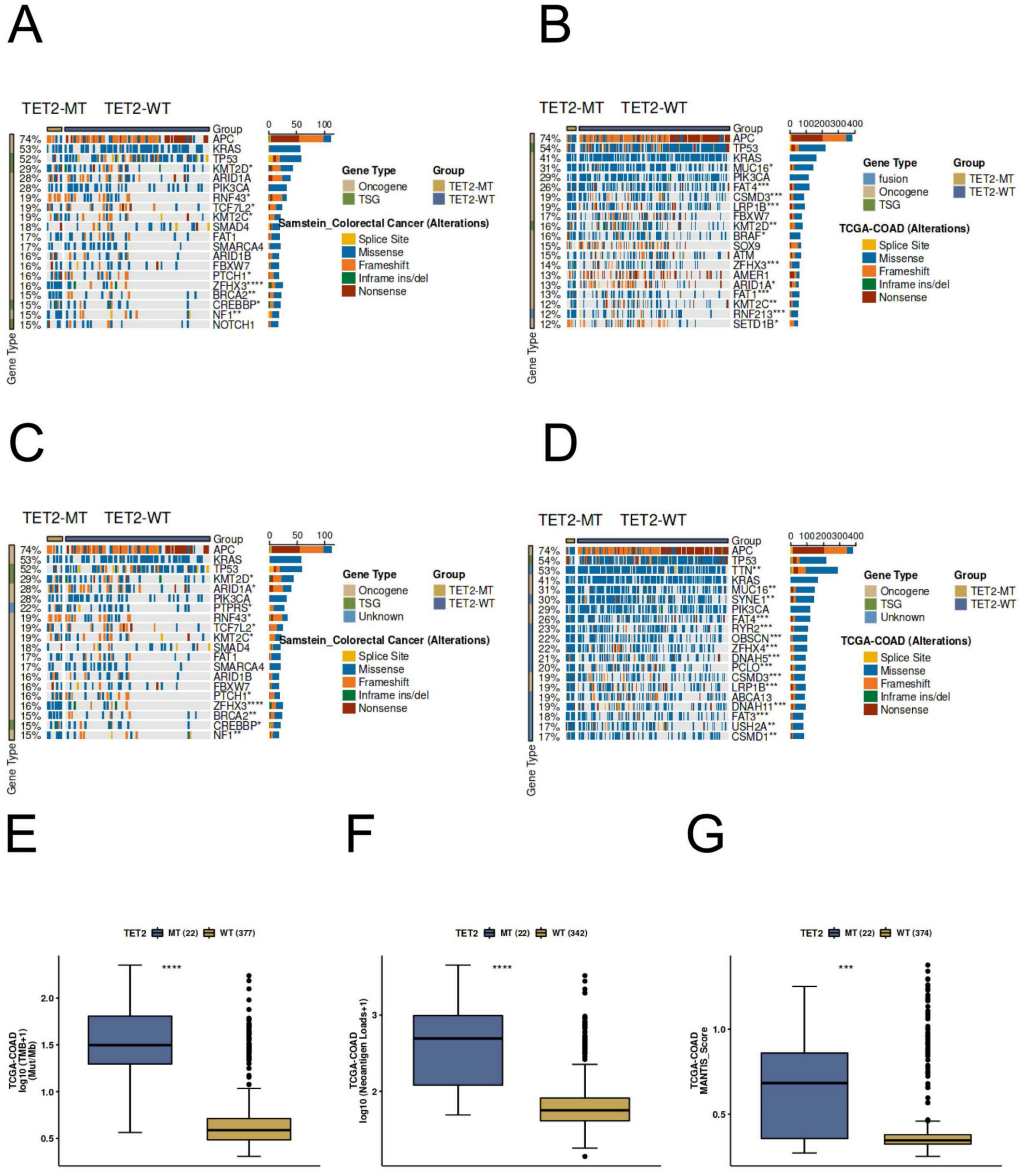
Genomic profiles of CRC patients. The Top 20 driver mutations in (A) the ICI-cohort and (B) the TCGA-CRC cohort. The Top 20 gene mutations in (C) the ICI-cohort and (D) the TCGA-CRC cohort. The association between TET2 mutant status and (E) TMB, (F) neoantigen loads, and (G) MANTIS score in TCGA-CRC cohort. WT=wild type; MT=mutant type.

Most of the gene mutation and driver mutation types in the ICI and TCGA cohorts are mainly missense mutations and frameshift mutations, and belong to an oncogene, and few belong to tumor suppressor genes (Figure 7A-7D). Importantly, the results of immunogenicity analysis indicated significantly higher TMB, neoantigen loads, and MANTIS score in TET2 mutant patients compared to the wild-type (Figure 7E-7G). In CRC patients, the TET2 mutant group exhibits a significantly higher MANTIS score, indicating that these samples are more closely associated with high microsatellite instability (MSI-H), which suggests a better response to immunotherapy. Overall, these results suggest that TET2 has the potential to serve as a predictive marker for the efficiency of CRC immunotherapy.

## Discussion

This study is the first to evaluate the causality and potential molecular mechanisms behind AD and CRC by combining MR analysis and multiple transcriptome analyses. We discovered a causal association between AD and a decreased risk of CRC. Moreover, we identified TET2 as a shared gene signature between AD and CRC. Notably, TET2 expression was found to be downregulated in both AD and CRC, and its downregulation was associated with a poorer prognosis in CRC. To enhance the robustness of our findings, additional independent datasets were utilized for validation, and immunohistochemical staining further supported the potential role of TET2 as a tumor suppressor gene in CRC. Methylation analysis revealed a significant correlation between high methylation of cg09666717 and cg12306086, downregulation of TET2, and poor prognosis in CRC.

Previous studies investigating the correlation between 'atopic conditions' and cancers have produced disparate findings 4-6. Two hypotheses link 'atopic conditions' and cancer: allergies stimulate the immune system, inhibiting cancer development, while chronic antigenic stimulation induces random prooncogenic mutations, increasing cancer risk 1, 2. Our study established a causal link between AD and decreased CRC risk, which was validated in an independent dataset. Prizment et al. reported that having 2 or more allergy-related conditions was associated with a decreased risk of CRC 10. Jacobs EJ et al. found that individuals who had both hay fever and asthma had a modestly lower risk of colorectal cancer mortality 19. Together with previous research, our findings support the "enhanced immune surveillance theory," indicating that stimulated immune systems better detect and eliminate malignant cells. Another possible explanation for this protective effect is that allergies serve as a defense mechanism against infestation by large parasites and environmental toxins. Consequently, individuals with heightened allergic reactions demonstrate enhanced capabilities in excreting and eliminating environmental toxins or carcinogens throughout their lifespan, in contrast to those with weaker reactions 20.

Understanding why AD patients are protected against CRC could be the key to uncovering novel treatments for both types of conditions. Through SMR analysis and transcriptome analyses, we identified TET2 as the shared hub gene between AD and CRC, with downregulation of TET2 being associated with a poorer prognosis in CRC. CRC growth is influenced by genetic and epigenetic abnormalities, with methylation at the 5-carbon of cytosines being a well-characterized epigenetic modification 21. This methylation often occurs in the promoter regions of tumor suppressor genes, leading to decreased expression and promoting cancer initiation and progression 22. DNA methylation can be actively removed through oxidative demethylation by the TET family of alphaglutarate-dependent oxygenases (TET1, TET2, and TET3). Specifically, TET enzymes catalyze the conversion of 5-methylcytosine to 5-hydroxymethylcytosine, facilitating DNA demethylation 23. In this study, analysis of differential methylation sites between CRC tumors and normal samples revealed high methylation of cg01210909, cg09666717, and cg12306086 in TET2. Importantly, the high methylation of cg09666717 and cg12306086 was significantly associated with poorer CRC prognosis. Therefore, we speculate that the downregulation of the tumor suppressor gene TET2 in CRC is associated with the hypermethylation of its sites cg09666717 and cg12306086, further leading to impaired demethylation function and facilitating the initiation and progression of CRC. Further experiments are needed to validate our results. Once confirmed, these results indicate that certain nuclear export inhibitors targeting TET2 may enhance the overall level of 5hmC by modulating TET2 in CRC cells, thereby promoting the active demethylation and expression of tumor suppressor genes. Additionally, inhibitors of DNA methyltransferases such as 5-azacytidine and 5-aza-2'-deoxycytidine may also potentially lead to the re-activation of the active DNA demethylation process, thereby facilitating the re-expression of TET2, offering a potential therapeutic approach for CRC 24.

GSEA revealed that high TET2 expression in both AD and CRC was associated with enrichment in 10 pathways related to the inflammatory response and immune regulation, including the intestinal immune network for IgA production, antigen processing and presentation, Th1 and Th2 cell differentiation, cell adhesion molecules, Th/17 cell differentiation, NOD-like receptor signaling pathway, C-type lectin receptor signaling pathway, sphingolipid signaling pathway, apoptosis, and NF-kappa B signaling pathway. Single-cell analysis further validated these findings by showing enrichment of the antigen processing and presentation and cell adhesion molecules pathways in B cells and the NOD-like receptor signaling pathway in macrophages. The NOD-like receptor (NLR) family plays a crucial role in immune responses, with diverse and intricate effects in CRC, potentially contributing to both pro-carcinogenic and anti-carcinogenic outcomes 25. For example, NLRP3 inflammasome has been found to hinder hepatic metastasis of CRC through IL-18 signaling 26, while increased expression of NLRP7 in CRC promotes M2 polarization of macrophages 27. Our results demonstrated that the enrichment of NOD-like receptor signaling in the TET2-high expression group, where its activation within macrophages correlates with improved prognosis in CRC. These findings suggest a potential association between NOD-like receptor signaling and the promotion of M1 polarization in macrophages in CRC, which could enhance the anti-tumor immune response. Further research is needed to validate these observations. Take together, our results shown that AD may reduce the risk of CRC by modulating various innate immune responses, such as antigen internalization and antigen presentation, adaptive immune responses, such as T-cell differentiation and T-cell activation, and inflammatory responses. Further research is needed to understand the specific mechanisms between AD and CRC.

Our study also revealed that TET2 is associated with immune infiltration in CRC, potentially contributing to its impact on prognosis. Specifically, TET2 expression levels show a significant negative correlation with the infiltration of M0 macrophages and activated mast cells. Macrophages can be classified into M1 and M2 subtypes 28. M1 macrophages are involved in inflammation and have antitumor immune properties 29, while M2 macrophages promote tumor development 30. M0 macrophages represent a transitional state without polarization toward M1 or M2 31. Within the tumor stroma, macrophages are known as tumor-associated macrophages 32. Tumor-associated macrophages often exhibit an M2 phenotype, which is closely associated with tumor angiogenesis and lymphangiogenesis and plays a crucial role in tumor initiation, progression, invasion, and metastasis 33. Li et al. found a significant enrichment of immunosuppressive angiogenesis pathways in CRC with high infiltration of activated mast cells. This infiltration was inversely correlated with CD8+ T-cell infiltration, suggesting that high activated mast cell infiltration in the CRC microenvironment may be associated with an immunosuppressive phenotype 34. Thus, the downregulation of TET2 expression in CRC may lead to an increased infiltration of immune suppressive cells, contributing to a poor prognosis. Another significant finding of this study is the predictive role of TET2 in CRC immunotherapy response. High immunogenicity is a critical factor in predicting the prognosis of patients undergoing immunotherapy. Numerous studies have demonstrated a strong association between elevated TMB and improved prognosis in patients receiving immunotherapy. Increased TMB can stimulate the generation of new neoantigens, which dendritic cells can process and present, leading to the maturation and activation of cytotoxic T cells. This process ultimately mediates the immune system's anti-tumor activity and enhances patients' response to treatment with immune checkpoint inhibitors 35. Our study revealed that CRC patients with TET2 mutations exhibited heightened immunogenicity, including elevated TMB, increased neoantigen loads, and a higher MANTIS score compared to the TET2-wild type group, suggesting patients' higher clinical benefit ratio from immunotherapy. Overall, these findings demonstrated that TET2 could be a potential prognostic biomarker and a novel therapeutic target for immune-related interventions in CRC.

This comprehensive study is the first to investigate the causal relationship and shared mechanisms between AD and CRC using multiple and complementary methods, shedding new light on the underlying biological processes of intrinsic immunity and CRC and providing potential therapeutic implications. However, there are certain limitations to consider. To address potential population stratification biases, our MR study focused on individuals of European ancestry. Therefore, caution should be exercised when generalizing these findings to other ancestries. Furthermore, our results of the shared mechanisms between AD and CRC remain at the level of data analysis. Additional molecular biology-based experiments are needed to validate our findings.

## Conclusions

In conclusion, our findings indicate a causal association between RA and a reduced risk of CRC. AD appears to modulate various innate and adaptive immune responses as well as inflammatory responses, thereby potentially reducing the risk of CRC. Interestingly, TET2 was identified as a shared gene signature between AD and CRC, with downregulation observed in both conditions. This gene may function as a tumor suppressor in CRC, and its low expression is associated with impaired demethylation and poor prognosis. Furthermore, TET2 may facilitate tumor-induced immune response activation and immune infiltration in CRC, thus exerting an anticancer effect. Further studies are necessary to elucidate the underlying mechanisms.

## Supplementary Material

Supplementary figure and tables.

## Abbreviations

AD: atopic dermatitis
CRC: colorectal cancer
MR: Mendelian randomization
SMR: Summary statistic data-based Mendelian randomization
IVs: instrumental variables
WGCNA: Weighted gene co-expression network analysis
GWAS: Genome-wide association study
IVW: inverse variance weighted
HEIDI: heterogeneity in dependent instruments
TCGA: The Cancer Genome Atlas
DEGs: differentially expressed genes
OS: overall survival
DSS: disease-specific survival
PFI: progression-free interval
KM: Kaplan‒Meier
TISCH2: Tumor Immune Single-cell Hub 2
GSEA: Gene set enrichment analysis
HPA: Human Protein Atlas
HRs: hazard ratios
OGT: O-linked N-acetylglucosamine transferase
TME: tumor immune microenvironment
RA: rheumatoid arthritis
