# Supplementary material for: Association between Atopic Dermatitis and Colorectal Cancer: TET2 as a Shared Gene Signature and Prognostic Biomarker
Source: J Cancer. 2024 Jan 20;15(5):1414–28. doi: 10.7150/jca.92238 (PMC10861813; doi:10.7150/jca.92238)

Supplementary Tables

Supplementary Table 1. Detailed information of the GWAS data used for MR analysis

First author	Datasets id	Reported trait	Sample Size	Case	Control	Population	Analysis
Fernandez-Rozadilla C	GCST90255675	Colorectal cancer	185,616	784,73	107,143	European	SMR Analysis
Huyghe JR	GCST012879	Colorectal cancer	32,072	19,948	12,124	European	Discovery outcome dataset
Mbatchou J	ebi-a-GCST90013866	Colorectal cancer	407,746	NA	NA	European	Validation outcome dataset
Sakaue S	ebi-a-GCST90018784	Atopic dermatitis	481,299	6,224	475,075	European	Validation exposure dataset
NA	finn-b-L12_ATOPIC	Atopic dermatitis	205,764	7,024	198,740	European	Discovery exposure dataset

Supplementary Table 2. Detailed information of the datasets used for bioinformatic analysis

GSE number	Platform	Samples	Tissue type	Disease	Analysis
GSE39582	GPL570	443 CRC and 19 CON	Tumor and mucosa	CRC	GSEA analysis
GSE113513	GPL15207	14 CRC and 14 CON	Tumor and mucosa	CRC	Immunoinfiltration analysis
GSE182740	GPL570	20 atopic dermatitis and 6 CON	Skin punch biopsies	Atopic dermatitis	Gene differential analysis
GSE121212	GPL16791	27 atopic dermatitis and 65 CON	Skin punch biopsies	Atopic dermatitis	Immunoinfiltration analysis and validation
GSE60709	GPL6947	19 atopic dermatitis and 14 CON	Skin punch biopsies	Atopic dermatitis	GSEA analysis

Supplementary Table 3. Results of MR analyses

Exposure	id. exposure	Outcome	id. outcome	Method	nSNP	OR (95%CI)	P-value	$P_{pleiotropy}$	$P_{heterogeneity}$
Discovery dataset									
Atopic dermatitis	finn-b-L12_ATOPIC	colorectal cancer	GCST012879	Inverse variance weighted	7	0.836 (0.748 - 0.936)	0.001782	0.440	0.289
Atopic dermatitis	finn-b-L12_ATOPIC	colorectal cancer	GCST012879	MR Egger	7	0.931 (0.707 - 1.226)	0.631714		
Atopic dermatitis	finn-b-L12_ATOPIC	colorectal cancer	GCST012879	Simple mode	7	0.871 (0.694 - 1.095)	0.269852		
Atopic dermatitis	finn-b-L12_ATOPIC	colorectal cancer	GCST012879	Weighted median	7	0.855 (0.744 - 0.984)	0.028751		
Atopic dermatitis	finn-b-L12_ATOPIC	colorectal cancer	GCST012879	Weighted mode	7	0.901 (0.733 - 1.107)	0.354861		
Validation dataset									
Atopic dermatitis	ebi-a-GCST90018784	colorectal cancer	ebi-a-GCST90013866	MR Egger	21	0.741 (0.605-0.908)	0.0093	0.071	0.279
Atopic dermatitis	ebi-a-GCST90018784	colorectal cancer	ebi-a-GCST90013866	Weighted median	21	0.885 (0.776-1.008)	0.06532		
Atopic dermatitis	ebi-a-GCST90018784	colorectal cancer	ebi-a-GCST90013866	Inverse variance weighted	21	0.885 (0.805-0.973)	0.01144		
Atopic dermatitis	ebi-a-GCST90018784	colorectal cancer	ebi-a-GCST90013866	Simple mode	21	0.800 (0.615-1.040)	0.11126		
Atopic dermatitis	ebi-a-GCST90018784	colorectal cancer	ebi-a-GCST90013866	Weighted mode	21	0.782 (0.655-0.935)	0.0137		

Supplementary Table 4. Results of SMR analysis

Gene	SYMBOL	ProbeChr	topSNP	A1	A2	Freq	Beta_SMR	SE_SMR	P_SMR	P_HEIDI
ENSG00000010327	STAB1	3	rs9855470	A	G	0.060636	0.13984	0.022741	7.78E-10	0.105
ENSG00000026036	RTEL1-TNFRSF6B	20	rs6062496	G	A	0.400596	-0.742158	0.159388	3.22E-06	0.0423
ENSG00000066933	MYO9A	15	rs12901580	A	C	0.204771	-0.192936	0.040336	1.73E-06	0.012
ENSG00000073605	GSDMB	17	rs12936231	C	G	0.485089	0.0387012	0.008782	1.05E-05	0.0159
ENSG00000078747	ITCH	20	rs1205344	A	G	0.493042	-0.183847	0.042202	1.32E-05	0.14
ENSG00000083838	ZNF446	19	rs3794971	C	T	0.219682	-0.279992	0.065267	1.79E-05	0.0519

ENSG00000089022	MAPKAPK5	12	rs79271898	T	C	0.081511	0.171498	0.036871	3.30E-06	0.116
ENSG00000099326	MZF1	19	rs3794964	C	T	0.263419	0.156866	0.028852	5.42E-08	0.011
ENSG00000099953	MMP11	22	rs11914035	T	C	0.101392	-0.349532	0.069585	5.08E-07	0.105
ENSG00000100311	PDGFB	22	rs5757573	C	T	0.38171	-0.100474	0.021834	4.19E-06	0.0261
ENSG00000101210	EEF1A2	20	rs6062486	G	A	0.296223	-0.542708	0.101731	9.57E-08	0.0462
ENSG00000101464	PIGU	20	rs6088552	G	A	0.420477	-0.414386	0.093994	1.04E-05	0.114
ENSG00000106077	ABHD11	7	rs13233747	A	G	0.355865	0.0787474	0.017635	7.99E-06	0.018
ENSG00000108107	RPL28	19	rs17700376	G	A	0.37674	-0.0608596	0.013774	9.94E-06	0.0462
ENSG00000111300	NAA25	12	rs78745958	T	C	0.082505	0.214583	0.046147	3.32E-06	0.426
ENSG00000114854	TNNC1	3	rs34332947	T	G	0.057654	-0.399409	0.073707	6.00E-08	0.0119
ENSG00000115073	ACTR1B	2	rs11692435	A	G	0.083499	-0.199068	0.029608	1.77E-11	0.198
ENSG00000116171	SCP2	1	rs1242331	A	G	0.347913	-0.0492476	0.010733	4.47E-06	0.301
ENSG00000121310	ECHDC2	1	rs1242331	A	G	0.347913	-0.0632144	0.01379	4.56E-06	0.511
ENSG00000122870	BICC1	10	rs10740734	G	A	0.486083	0.221104	0.048557	5.27E-06	0.0354
ENSG00000123810	B9D2	19	rs4803457	T	C	0.38171	-0.376177	0.059501	2.58E-10	0.0505
ENSG00000124762	CDKN1A	6	rs12199346	A	C	0.208748	-0.288479	0.04276	1.51E-11	0.0337
ENSG00000125686	MED1	17	rs12450559	G	A	0.250497	0.239263	0.052408	4.99E-06	0.0714
ENSG00000125845	BMP2	20	rs6140415	A	G	0.154076	0.324046	0.041498	5.77E-15	0.0412
ENSG00000127837	AAMP	2	rs13003334	T	A	0.412525	0.644892	0.098861	6.88E-11	0.0531
ENSG00000130724	CHMP2A	19	rs3794964	C	T	0.263419	0.593162	0.127682	3.39E-06	0.553
ENSG00000130725	UBE2M	19	rs3794964	C	T	0.263419	0.304709	0.062902	1.27E-06	0.0858
ENSG00000131969	ABHD12B	14	rs17123107	G	A	0.203777	-0.113052	0.02424	3.10E-06	0.343
ENSG00000133030	MPRIP	17	rs11867934	T	C	0.191849	0.358146	0.084972	2.50E-05	0.626
ENSG00000135862	LAMC1	1	rs11588675	T	C	0.426441	0.134403	0.01334	7.14E-24	0.381
ENSG00000136280	CCM2	7	rs10951794	A	G	0.207753	-0.0845615	0.015138	2.32E-08	0.0272
ENSG00000137834	SMAD6	15	rs76912608	T	C	0.250497	0.512029	0.091023	1.85E-08	0.103
ENSG00000140307	GTF2A2	15	rs6151590	A	G	0.355865	0.103072	0.022612	5.16E-06	0.0616
ENSG00000142039	CCDC97	19	rs2241715	A	C	0.314115	-0.682785	0.128381	1.05E-07	0.165
ENSG00000143344	RGL1	1	rs1184639	C	G	0.365805	-0.145762	0.029376	6.98E-07	0.046
ENSG00000146833	TRIM4	7	rs2572010	C	T	0.468191	-0.0560289	0.010736	1.80E-07	0.078
ENSG00000161395	PGAP3	17	rs2952152	T	C	0.318091	0.110543	0.022272	6.93E-07	0.0855
ENSG00000161405	IKZF3	17	rs907091	T	C	0.489066	-0.09225	0.020548	7.14E-06	0.478
ENSG00000162704	ARPC5	1	rs2767304	G	A	0.400596	-0.075644	0.013681	3.22E-08	0.547
ENSG00000165171	METTL27	7	rs8629	T	C	0.274354	-0.0480776	0.008883	6.22E-08	0.259
ENSG00000168769	TET2	4	rs11729069	G	C	0.163022	-0.686382	0.158771	1.54E-05	0.021
ENSG00000174851	YIF1A	11	rs2155030	G	A	0.173956	-0.21946	0.051741	2.22E-05	0.0447
ENSG00000175711	B3GNTL1	17	rs9890743	T	C	0.296223	-0.101005	0.018528	4.99E-08	0.0349
ENSG00000177150	FAM210A	18	rs77564907	A	G	0.15507	0.271818	0.06406	2.20E-05	0.738
ENSG00000179295	PTPN11	12	rs12425405	C	G	0.082505	0.254444	0.05863	1.43E-05	0.0576
ENSG00000179921	GPBAR1	2	rs11677953	A	G	0.366799	-0.251128	0.03275	1.75E-14	0.0174
ENSG00000183520	UTP11	1	rs4360494	G	C	0.471173	0.235382	0.031913	1.63E-13	0.108
ENSG00000189143	CLDN4	7	rs1989670	T	A	0.274354	-0.330597	0.070076	2.39E-06	0.181

ENSG00000197093	GAL3ST4	7	rs11764176	T	G	0.26839	-0.144762	0.0343	2.44E-05	0.835
ENSG00000198270	TMEM116	12	rs4767068	G	A	0.167992	0.0938929	0.02024	3.50E-06	0.576
ENSG00000198324	PHETA1	12	rs11065884	G	A	0.224652	0.276074	0.061125	6.29E-06	0.823
ENSG00000203999	LINC01270	20	rs1971480	G	T	0.311133	0.14137	0.029135	1.22E-06	0.0106
ENSG00000204147	ASAH2B	10	rs2820760	A	G	0.162028	-0.681977	0.158217	1.63E-05	0.32
ENSG00000204644	ZFP57	6	rs416568	A	T	0.246521	0.0410055	0.008607	1.89E-06	0.0698
ENSG00000213533	STIMATE	3	rs1986656	T	C	0.117296	-0.18383	0.030666	2.04E-09	0.0457
ENSG00000224397	PELATON	20	rs3761181	A	G	0.459245	-0.0733298	0.012975	1.59E-08	0.692
ENSG00000226469	ADAM1B	12	rs7134084	A	G	0.16501	0.113603	0.024782	4.56E-06	0.129
ENSG00000226979	LTA	6	rs2071590	A	G	0.363817	-0.267616	0.046571	9.12E-09	0.0374
ENSG00000228789	HCG22	6	rs1265054	C	T	0.514911	-0.0689083	0.016316	2.41E-05	0.014
ENSG00000229186		12	rs12423572	C	A	0.082505	-0.0684236	0.014561	2.61E-06	0.844
ENSG00000230795	HLA-K	6	rs416568	A	T	0.246521	-0.0460454	0.009699	2.06E-06	0.133
ENSG00000232810	TNF	6	rs1121800	A	T	0.404573	-0.166747	0.026172	1.87E-10	0.166
ENSG00000233077	LINC01271	20	rs4811018	A	G	0.312127	0.21773	0.048143	6.11E-06	0.0454
ENSG00000234608	MAPKAPK5-AS1	12	rs16941759	A	G	0.16501	0.104945	0.021916	1.68E-06	0.0841
ENSG00000239732	TLR9	3	rs13098856	A	G	0.060636	0.871861	0.19794	1.06E-05	0.125
ENSG00000258398		14	rs11157782	T	C	0.202783	-0.057633	0.01224	2.50E-06	0.791
ENSG00000260997		7	rs6963832	T	C	0.095427	-0.0507809	0.00962	1.30E-07	0.0332
ENSG00000261338		2	rs736730	C	T	0.412525	0.0604115	0.007672	3.43E-15	0.0435
ENSG00000261716	H2BC20P	1	rs7531664	C	T	0.134195	-0.112835	0.026849	2.64E-05	0.327
ENSG00000262820		16	rs11075687	T	C	0.214712	0.191731	0.043824	1.21E-05	0.121
ENSG00000269202		20	rs633198	C	T	0.483101	0.057938	0.013268	1.26E-05	0.0294

Supplementary Table 5. Results of GSEA exploring the shared pathways involving TET2 in CRC

ID	Description	P_ value	P. adjust	q_value
hsa03030	DNA replication	0.001	0.001	0.008
hsa04977	Vitamin digestion and absorption	0.001	0.001	0.008
hsa04975	Fat digestion and absorption	0.001	0.001	0.009
hsa03008	Ribosome biogenesis in eukaryotes	0.002	0.002	0.010
hsa04310	Wnt signaling pathway	0.003	0.003	0.014
hsa00100	Steroid biosynthesis	0.003	0.003	0.015
hsa03020	RNA polymerase	0.008	0.008	0.034
hsa04974	Protein digestion and absorption	0.011	0.011	0.042
hsa03430	Mismatch repair	0.013	0.013	0.044
hsa00030	Pentose phosphate pathway	0.020	0.020	0.060
hsa00120	Primary bile acid biosynthesis	0.024	0.024	0.064
hsa00330	Arginine and proline metabolism	0.025	0.025	0.065
hsa01230	Biosynthesis of amino acids	0.027	0.027	0.067
hsa03420	Nucleotide excision repair	0.032	0.032	0.074
hsa01200	Carbon metabolism	0.033	0.033	0.074

Supplementary Table 6. Results of GSEA exploring the shared pathways involving TET2 in AD

ID	Description	P_ value	P. adjust	q_value
hsa00592	alpha-Linolenic acid metabolism	<0.0001	<0.0001	0.003
hsa05310	Asthma	<0.0001	<0.0001	0.003
hsa05332	Graft-versus-host disease	<0.0001	<0.0001	0.003
hsa05330	Allograft rejection	<0.0001	<0.0001	0.003
hsa04940	Type I diabetes mellitus	<0.0001	<0.0001	0.003
hsa05321	Inflammatory bowel disease	<0.0001	<0.0001	0.003
hsa05322	Systemic lupus erythematosus	<0.0001	<0.0001	0.003
hsa04672	Intestinal immune network for IgA production	<0.0001	<0.0001	0.003
hsa05320	Autoimmune thyroid disease	<0.0001	<0.0001	0.003
hsa05416	Viral myocarditis	<0.0001	<0.0001	0.003
hsa04612	Antigen processing and presentation	<0.0001	<0.0001	0.003
hsa04640	Hematopoietic cell lineage	<0.0001	<0.0001	0.003
hsa04658	Th1 and Th2 cell differentiation	<0.0001	<0.0001	0.003
hsa05145	Toxoplasmosis	<0.0001	<0.0001	0.003
hsa04142	Lysosome	<0.0001	<0.0001	0.003
hsa04145	Phagosome	<0.0001	<0.0001	0.003
hsa04514	Cell adhesion molecules	<0.0001	<0.0001	0.003
hsa05152	Tuberculosis	<0.0001	<0.0001	0.003
hsa05164	Influenza A	<0.0001	<0.0001	0.003
hsa05169	Epstein-Barr virus infection	<0.0001	<0.0001	0.003
hsa05150	Staphylococcus aureus infection	<0.0001	<0.0001	0.005
hsa04664	Fc epsilon RI signaling pathway	0.001	0.001	0.014
hsa04659	Th17 cell differentiation	0.001	0.001	0.014
hsa00051	Fructose and mannose metabolism	0.002	0.002	0.016
hsa05140	Leishmaniasis	0.002	0.002	0.016
hsa05323	Rheumatoid arthritis	0.003	0.003	0.028
hsa00190	Oxidative phosphorylation	0.004	0.004	0.035
hsa05146	Amoebiasis	0.007	0.007	0.050
hsa04966	Collecting duct acid secretion	0.008	0.008	0.055
hsa04621	NOD-like receptor signaling pathway	0.010	0.010	0.063
hsa04625	C-type lectin receptor signaling pathway	0.010	0.010	0.064
hsa04071	Sphingolipid signaling pathway	0.012	0.012	0.073
hsa04210	Apoptosis	0.012	0.012	0.073
hsa04512	ECM-receptor interaction	0.016	0.016	0.090
hsa03050	Proteasome	0.018	0.018	0.094
hsa00565	Ether lipid metabolism	0.019	0.019	0.102
hsa00591	Linoleic acid metabolism	0.021	0.021	0.109
hsa04650	Natural killer cell mediated cytotoxicity	0.026	0.026	0.131
hsa04064	NF-kappa B signaling pathway	0.028	0.028	0.133
hsa04974	Protein digestion and absorption	0.028	0.028	0.133

hsa04136	Autophagy - other	0.032	0.032	0.148
hsa05133	Pertussis	0.035	0.035	0.160
hsa04666	Fc gamma R-mediated phagocytosis	0.038	0.038	0.167
hsa04140	Autophagy - animal	0.040	0.040	0.171
hsa04370	VEGF signaling pathway	0.042	0.042	0.172
hsa04622	RIG-I-like receptor signaling pathway	0.042	0.042	0.172
hsa00100	Steroid biosynthesis	0.044	0.044	0.172
hsa00564	Glycerophospholipid metabolism	0.049	0.049	0.174
hsa00830	Retinol metabolism	0.049	0.049	0.174

Figure S1. Gene set enrichment analysis of TET2. (A) GSEA results of TET2 in CRC. (B) GSEA results of TET2 in atopic dermatitis.

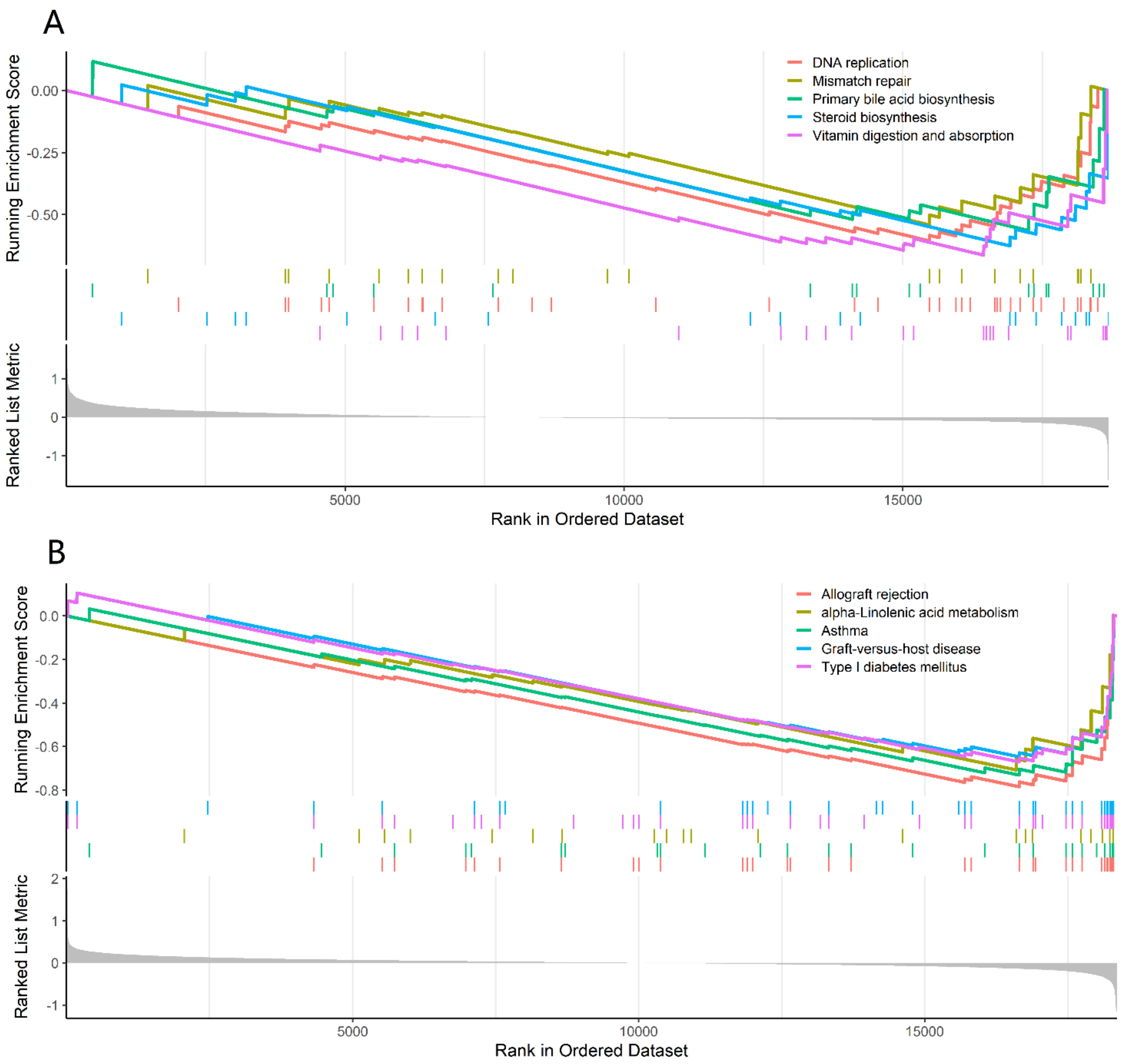

Supplement: Supplementary file 1 — Supplementary figure and tables. [file jcav15p1414s1.pdf]
